# Cortical Morphology Differences in Subjects at Increased Vulnerability for Developing a Psychotic Disorder: A Comparison between Subjects with Ultra-High Risk and 22q11.2 Deletion Syndrome

**DOI:** 10.1371/journal.pone.0159928

**Published:** 2016-11-09

**Authors:** Geor Bakker, Matthan W. A. Caan, Wilhelmina A. M. Vingerhoets, Fabiana da Silva- Alves, Mariken de Koning, Erik Boot, Dorien H. Nieman, Lieuwe de Haan, Oswald J. Bloemen, Jan Booij, Thérèse A. M. J. van Amelsvoort

**Affiliations:** 1 Department of Psychiatry & Psychology, University of Maastricht, Maastricht, The Netherlands; 2 Department of Nuclear Medicine, Academic Medical Center, University of Amsterdam, Amsterdam, The Netherlands; 3 Department of Radiology, Academic Medical Center, University of Amsterdam, Amsterdam, The Netherlands; 4 Department of Psychiatry, Academic Medical Center, University of Amsterdam, Amsterdam, The Netherlands; 5 GGZ Centraal, Center for Mental Health Care Innova, Amersfoort, The Netherlands; 6 Arkin Mental Health Care, Amsterdam, The Netherlands; 7 The Dalglish Family 22q Clinic, Toronto, Ontario, Canada; Chiba Daigaku, JAPAN

## Abstract

**Introduction:**

Subjects with 22q11.2 deletion syndrome (22q11DS) and subjects with ultra-high risk for psychosis (UHR) share a risk of approximately 30% to develop a psychotic disorder. Studying these groups helps identify biological markers of pathophysiological processes involved in the development of psychosis. Total cortical surface area (cSA), total cortical grey matter volume (cGMV), cortical thickness (CT), and local gyrification index (LGI) of the cortical structure have a distinct neurodevelopmental origin making them important target markers to study in relation to the development of psychosis.

**Materials and Methods:**

Structural T1-weighted high resolution images were acquired using a 3 Tesla Intera MRI system in 18 UHR subjects, 18 22q11DS subjects, and 24 matched healthy control (HC) subjects. Total cSA, total cGMV, mean CT, and regional vertex-wise differences in CT and LGI were assessed using FreeSurfer software. The Positive and Negative Syndrome Scale was used to assess psychotic symptom severity in UHR and 22q11DS subjects at time of scanning.

**Results:**

22q11DS subjects had lower total cSA and total cGMV compared to UHR and HC subjects. The 22q11DS subjects showed bilateral lower LGI in the i) prefrontal cortex, ii) precuneus, iii) precentral gyrus and iv) cuneus compared to UHR subjects. Additionally, lower LGI was found in the left i) fusiform gyrus and right i) pars opercularis, ii) superior, and iii) inferior temporal gyrus in 22q11DS subjects compared to HC. In comparison to 22q11DS subjects, the UHR subjects had lower CT of the insula. For both risk groups, positive symptom severity was negatively correlated to rostral middle frontal gyrus CT.

**Conclusion:**

A shared negative correlation between positive symptom severity and rostral middle frontal gyrus CT in UHR and 22q11DS may be related to their increased vulnerability to develop a psychotic disorder. 22q11DS subjects were characterised by widespread lower degree of cortical gyrification linked to early and postnatal neurodevelopmental pathology. No implications for early neurodevelopmental pathology were found for the UHR subjects, although they did have distinctively lower insula CT which may have arisen from defective pruning processes during adolescence. Implications of these findings in relation to development of psychotic disorders are in need of further investigation in longitudinal studies.

## Introduction

Patients with psychotic disorders, with schizophrenia being the most severe form, can present with positive symptoms such as hallucinations, delusions, and disturbed thoughts, cognitive dysfunction, and affective dysregulation. To date, little is known about the underlying mechanisms of psychotic disorders. Structural magnetic resonance imaging (MRI) studies investigate aspects of the cortical morphology to identify changes in neurodevelopmental trajectories related to the development of psychotic symptoms. Different aspects of the cortical architecture can be traced back to prenatal cortical formation and postnatal maturation processes. A body of structural MRI work done in psychotic disorders report signs of fronto-temporal cortical atrophy and atypical frontal gyrification [1,2]. Investigating indices of cortical morphology in subjects with increased vulnerability to develop psychotic symptoms may identify biomarkers related to the onset of psychotic disorders.

Subjects with 22q11.2 deletion syndrome (22q11DS) have a 30% lifetime risk to develop a psychotic disorder [3]. The syndrome is caused by an interstitial micro deletion at the 22q11.2 locus, which codes, at least in part, for genes involved in axonal migration making these subjects a unique population to study the influence of diminished gene expression on cortical morphology maturation in relation to psychosis [4–7]. Subjects with ultra-high risk (UHR) for psychosis equally have a 30% risk develop a psychotic disorder within 2 years after identification [8]. Subjects are identified as being at UHR if they are help seeking and present clinically with 1) attenuated psychotic symptoms (APS), and/or 2) brief psychotic symptoms which spontaneously remit within a week (BLIPS), and/or 3) a schizotypal personality disorder or a first-degree relative with a psychotic disorder, in combination with a significant decrease in functioning during the previous year [9].

Total cortical surface area (cSA), cortical gray matter volume (cGMV), cortical thickness (CT), and the local gyrification index (LGI) (complexity of gyral patterning) are indices describing different aspects of the cortical architecture, and can be assessed in the living brain using MRI. The cortical structure is largely completed during prenatal development, with distinct maturational milestones related to each index. Early prenatal cortical formation is characterised by increased CT and cGMV, and later maturation by further expansion of the cSA by increased folding, or gyrification [10]. Postnatal maturation of the cortical layer involves increased gyrification of the superior and inferior frontal gyri. Additionally, during adolescence pruning of inefficient neurons takes place affecting CT measures [11]. Each measure thus is the manifestation of a distinct underlying neurodevelopmental mechanism- both prenatal and postnatal, and therefore, of interest to study in relation to psychosis [11].

To date, there are few studies investigating cortical morphology in 22q11DS, with none solely investigating adults. Studies in adolescent and adult 22q11DS subjects report lower CT of the superior temporal gyrus and cingulate cortex in comparison to HC [12]. Additionally, a longitudinal study identified greater CT loss in the prefrontal cortex over time, likely through abnormal pruning [13]. However, the cortical morphology of 22q11DS subjects is much more characterised by lower cSA and widespread hypogyrification [12,14,15]. Hypogyrification has been found throughout anterior and posterior midline cerebral cortex, orbitofrontal cortex, frontal pole, angular gyrus, inferior parietal, mid-central and post central gyrus in adolescent and early adult 22q11DS subjects compared to HC [12]. Similar regions with lower LGIs were found when comparing adolescent and early adult 22q11DS subjects to non-clinical, otherwise healthy subjects with psychotic symptoms, suggesting this may be an specific endophenotype of 22q11DS [12]. Interestingly, this study identified shared lower CT of the superior temporal gyrus between the 22q11DS subjects and non-clinical subjects with psychotic symptoms, showing the most pronounced reductions in 22q11DS subjects [12]. In younger 22q11DS subjects lower LGI of the frontal cortex was found associated with increased psychotic symptom severity [13,14], although this is ill investigated in adults.

Several cross-sectional studies investigating adult UHR subjects of 20 years of age and onwards, have shown thinner CT in the anterior cingulate cortex (ACC), medial and superior temporal lobe, ventromedial prefrontal cortex and parahippocampal gyri [16–18]. The degree of cortical thinning was also related to increased psychotic symptom severity in these UHR subjects [16], although no differential thinning in these areas between familial and non-familial UHR subjects was reported, pointing towards non-inherited factors affecting CT maturation [17,18]. These studies also identified similar, although more exuberated, thinning in these regions in first episode psychosis patients [17–19]. In non-familial UHR subjects no aberrant gyral patterning has been reported [19,20], although hypogyria in the ACC was found to be hereditable between schizophrenia probands and unaffected siblings [20].

Thus, the body of research assessing cortical maturation to date identifies disparate cortical morphology in UHR and 22q11DS subjects, but potentially also signs of overlapping anomalies. These shared anomalies seem to centre on CT maturation of frontal-temporal regions. However, a limited number of studies have assessed LGI in UHR subjects, and no study has compared subjects meeting the UHR criteria with 22q11DS subjects. Additionally, little investigation linking cortical morphology aberrations to psychotic symptoms have been conducted. Therefore, the aim of the current study was to compare indices of cortical architecture in UHR and 22q11DS subjects and how these are associated with psychotic symptom severity. Outcomes of this study may help to identify specific clinical and genetic biomarkers for psychotic disorders.

## Materials and Methods

The current study included 18 UHR subjects, 18 22q11DS subjects, and 24 age and gender matched healthy control (HC) subjects. Of the 18 22q11DS subjects, 8 had a history of a psychotic disorder. Demographics are displayed in Table 1. There were no significant between-group differences in age and gender in the UHR, 22q11DS, and HC groups. For the two at risk groups, psychotic symptom severity was assessed using the Positive and Negative Syndrome Scale (PANSS)[21]. UHR subjects scored significantly higher on the PANSS positive symptom and general psychopathology subscale compared to 22q11DS subjects. All UHR subjects were antipsychotic naïve. The UHR subjects sample included 16 subjects with APS, 2 with BLIPS, and none meeting the criteria for familial risk. All 22q11DS subjects that had a history of psychosis were on antipsychotic treatment, the others were antipsychotic naïve. No significant differences in PANSS scores were found between the 22q11DS subjects with an history of psychosis and those without on all PANSS subscales at time of scanning (PANSS positive: t = 1.92, p = 0.0725, PANSS negative: t = 1.91, p = 0.075, PANSS general: t = 1.91, p = 0.085). None of the 22q11DS subjects had a parent with a confirmed deletion. The study was approved by an independent Medical Ethics Committee of the Academic Medical Centre (AMC) in Amsterdam. All subjects gave written informed consent after all study procedures were fully explained.

**Table 1:** Demographic and clinical variables.

	*22q11DS (n = 18)*	*UHR (n = 18) (APS*:*16/BLIPS*:*2 /Fam*: *0)*	*Controls (n = 24)*	*stats*	*p*
Age (sd) in years	25 (2.6)	22.7 (3.6)	23.4 (3.2)	F = 2.42	0.09
Gender male/female	9/9	8/10	14/10	X^2^ = 0.82	0.66
PANSS positive (M/sd)	8.8 (2.6)	11.3 (2.4)		t = 2.92	0.006
PANSS negative (M/sd)	12.6 (4.6)	11.8 (5.7)		t = 0.48	0.63
PANSS general (M/sd)	25.3 (7.1)	45.1 (12.1)		t = 6.02	0.001
Psychosis yes/no	8/10				
Antipsychotic medication (yes/no)	8/10				
Congenital heart disease yes/no	8/10				

**PANSS**: Positive and Negative Syndrome Scale; **UHR**: ultra-high risk subjects; **22q11DS**: 22q11.2 deletion syndrome subjects; **APS**: attenuated psychotic symptoms; **BLIPS:** brief limited psychotic symptoms; **Fam:** first degree relative with psychotic disorder; **M**: mean; **sd**: standard deviation.

The UHR subjects were recruited through an expert program for early detection of psychosis at the AMC, and diagnosed by a trained psychiatrist or psychologist using the structured interview for prodromal syndromes (SIPS) [22]. Age range for inclusion was set between 18–30 years. Individuals with 22q11DS were recruited through the Dutch 22q11DS family association and several Dutch centres for clinical genetics. All 22q11DS diagnoses were molecularly confirmed. HC subjects were recruited by means of advertisement, and matched for age, and gender. HC subjects were excluded if they were diagnosed with a mental disorder, or had a positive family history for psychotic disorders. Additional exclusion criteria for all subjects were; present substance use or history of abuse or dependency, neurological affliction, or pregnancy. All study participants participated in other studies [23–25].

### Structural MR data acquisition

Whole brain MRI acquisition took place at the Department of Radiology (AMC, Amsterdam, The Netherlands) using a 3 Tesla Intera MRI system (Philips, Best, The Netherlands) equipped with a 6 channel sense head coil. Structural 3D T1-weighted high resolution, gradient echo images were acquired; full head coverage; repetition time (TR)/ echo time (TE) of 9.8/4.6 ms; axial orientation; 120 continuous (no inter-slice gap) slices; slice thickness 1.2 mm; flip angle 8°; 224×117 mm field of view (FOV); acquisition matrix 192×152×120; acquisition voxel size 1.17×1.17×1.20 mm.

### Calculation of cSA, cGMV, CT and LGI

cSA, cGMV, CT and LGI were measured using FreeSurfer version 5.3 software. FreeSurfer uses a surface based imaging processing pipeline to reconstruct the brain’s cortical surface from structural MRI data. Details are extensively described elsewhere [26–29]. In short, per subject, image intensity is normalised to account for magnetic field in homogeneity and the skull and other non-brain tissue are removed, after which a unitary white matter volume is created using a connected components algorithm. This is used as starting point for the initial grey–white surface. This surface is then covered with a polygonal tessellation and smoothed resulting in high-resolution vertices over both cerebral hemispheres. Using a deformable surface algorithm, the surface is further expanded to the grey- cerebrospinal fluid interface–or pial surface. For all subjects, the cortical surface model was checked.

From these two surfaces, cSA, cGMV, CT, and LGI were calculated. CT was quantified by the distance between the pial surface and gray-white matter junction. The cSA was quantified by calculating the average triangular size surrounding the tessellated cortical vertices of the pial surface. Total cGMV was calculated by multiplying cSA and CT at each vertex. LGI was calculated according to the method of Schaer and co-workers [30], implemented in FreeSurfer to assess local gyral complexity. LGI is defined as the ratio of amount buried cortex within the sulcal folds to visible cortex in circular regions of interest. The cortex grows primarily through radial expansion making this method sensitive to identifying early defects in cortical development. Using a morphological closing operation the outer surface was computed from the pial surface. A circular region of interest was then delineated on this outer surface and its corresponding region of interest on the pial surface identified using a validated matching algorithm [30].

### Statistical analyses

Statistical analyses were conducted using SPSS release 20 for Windows (SPSS Inc. Chicago, IL, USA). Group differences in age and PANSS scores were examined using three groups analysis of variance (ANOVA). Group differences in gender were tested using Chi-square tests. Level of statistical significance was set at p<0.05 (two tailed). Global differences between groups in cortical morphology indices cSA, cGMV, and mean CT, were assessed using an ANOVA with post-hoc Bonferroni tests to investigate group effects driving these differences. Additionally, a sub- analysis was conducted assessing these global cortical morphology indices in the 22q11DS subjects with a history of psychosis to those without.

### Vertex-wise SA, CT, and local GI calculations

Regional morphometric differences in SA and CT were assessed between groups using a vertex-by- vertex general linear model (GLM) controlling for linear and non-linear effects of age, gender and brain segmentation volume (BSV) using FreeSurfer’s GUI-based QDEC (Query, Design, Estimate, Contrast) interface [31]. 22q11DS subjects are characterised by smaller brains [32–34] and therefore, BSV was controlled for in the vertex-wise analysis. The cortical surfaces from each participant were transformed to an average template surface space and maps were created using statistical thresholds of p = 0.05, and were smoothed at a full width half maximum (FWHM) level of 10mm. Statistical analysis of CT and LGI were performed at 160,000 vertices per hemisphere, and these maps were false discovery rate (FDR) corrected using a threshold of p = 0.05 using 10.000 iterations. Post-hoc pairwise t-tests were conducted to determine which group differences were driving the main effects. An additional GLM was run between the 22q11DS subjects with and without a history of a psychotic disorder to investigate changes in CT and LGI related to the development of psychosis. Age and BSV were found to show a linear variation with CT and thus, despite group matching, added as nuisance regressors in the model. A correlation between psychotic symptom severity and morphology was assessed using a GLM in 22q11DS and UHR subjects, controlling for age, gender and BSV. Presence of congenital heart disease in 22q11DS subjects has been associated with higher CT and lower LGI measures [13,14,35]. Therefore, interactions of congenital heart disease on LGI and CT were checked at each vertex (CT) and cortical point (LGI) within the 22q11DS group. Again a sub-analysis was conducted between 22q11DS subjects with and without a history of a psychotic disorder.

## Results

### Global morphology; cSA, cGMV, and mean CT

**Fig 1:**
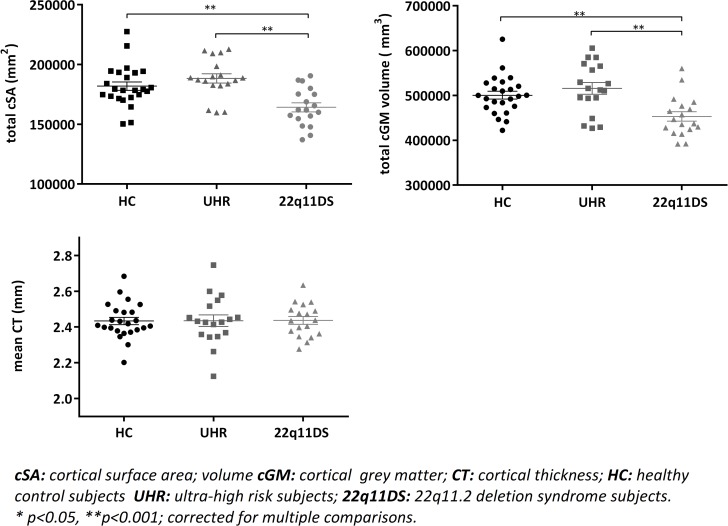
Total cSA, total cGMV and mean CT per group. Plots show that 22q11DS subjects had significantly lower total cSA and cGMV compared to UHR and HC. No between-group differences were found in mean CT. UHR subjects had comparable measures of cGMV, total cSA and average CT compared to HC. See S1, S2, S3 and S4 Files.

Analyses showed significantly lower cSA and cGMV in subjects with 22q11DS compared to UHR subjects (cSA: t = 4.38, p<0.001; cGMV: t = 3.93, p<0.001) and HC subjects (cSA: t = 3.423, p = 0.001; cGMV: t = 3.19, p = 0.001). The UHR subjects did not differ significantly from HC in cSA (t = 1.26, p = 0.22) and cGMV (t = 1.011, p = 0.33). No group differences were found in mean CT (F = 0.003 (59), p = 0.22) between 22q11DS, UHR, and HC subjects. Results are summarised in Fig 1. In a sub-analysis, no significant differences were found in cSA, cGMV, and mean CT between 22q11DS subjects with and without history of a psychotic disorder.

### Vertex-wise comparisons cSA and CT

No significant regional differences in cSA were found between UHR and 22q11, nor between each risk group and HC subjects. 22q11DS subjects did have significantly increased CT in the insula compared to UHR subjects (p<0.05, FDR-corrected). In comparison to HC, they had bilateral increased CT in the lingual gyrus and precuneus (p<0.05, FDR-corrected). No corrected regional differences were found in CT between the UHR and HC subjects. In a sub-analysis no significant differences were found in CT between 22q11DS subjects with a history of a psychotic disorder in comparison to those without.

### Comparisons in LGI

**Fig 2:**
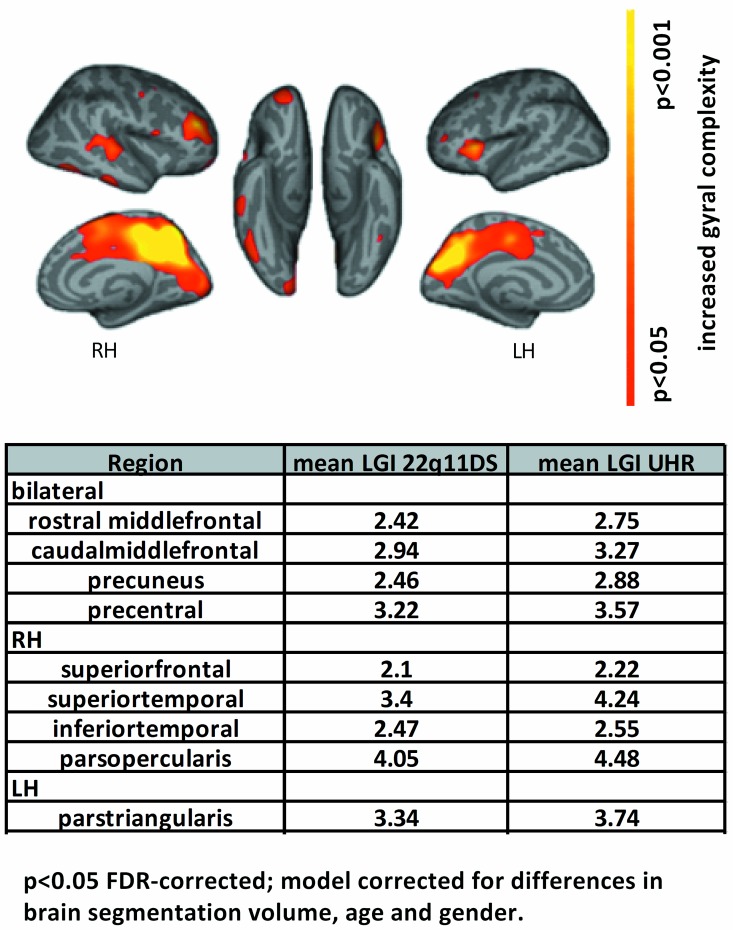
Vertex-wise comparisons showing effect of diagnosis on local gyrification index (LGI) between 18 UHR and 18 age- and gender matched 22q11DS subjects. Comparisons show bilaterally lower gyral complexity in 22q11DS compared to UHR subjects. Significant clusters and corresponding LGI are displayed in the table. Note that LGI indices range between 1 and 5, with higher numbers denoting higher gyral complexity. RH: right hemisphere; LH: left hemisphere. See S1 and S2 Files.

Compared to the UHR subjects, the 22q11DS subjects had lower LGI values: i) bilaterally in the middle frontal gyrus (caudal and rostral), precentral gyrus, precuneus, and cuneus ii), in the right inferior and superior temporal gyrus, superior frontal gyrus, par operculum, and pericalcarine and iii) in the left pars triangularis, and fusiform gyrus. Regions with significantly lower LGIs in 22q11DS compared to UHR are shown in Fig 2. Compared to HC, 22q11DS subjects had lower GI values in the left rostral middle frontal gyrus, medial orbitofrontal inferior temporal gyrus, and right pars orbitalis, middle temporal and paracentral gyrus. 22q11DS subjects with psychosis and without psychosis showed no significant differences in LGIs measures. No significant effect of congenital heart pathology on CT and LGI measures were found the subjects with 22q11DS. When comparing UHR and HC, no significant differences in LGIs were found.

### Correlations between PANSS scores and morphometric indices

A significant negative correlation between rostral middle frontal gyrus CT and positive symptom severity was found in UHR and 22q11DS subjects (p<0.05, FDR-corrected; R^2^ RH = -5.11; R^2^ LH = -3.74). When accounting for group, no significant correlation between rostral middle frontal gyrus CT and positive symptom severity was found. No significant correlation was found between any of the other morphometric indices and scores on the negative symptom subscale or general psychopathology subscale of the PANSS.

## Discussion

The current study is the first explorative study to compare and contrast indices of structural cortical maturation in adults with (non-familial) UHR and 22q11DS, and to evaluate these findings in relation to severity of psychotic symptoms. Key findings showed that 22q11DS subjects were characterized by lower cSA and cGMV compared to UHR and HC subjects. Moreover, the 22q11DS subjects were characterized by lower LGIs in the prefrontal cortex, temporal lobe, precuneus and cuneus in comparison to both UHR and HC subjects. In addition to these regions, 22q11DS subjects had lower LGI of the precentral and fusiform gyrus in comparison to UHR subjects. Results also revealed lower CT of the rostral middle frontal gyrus was related to increased positive symptom severity in both risk profiles. In comparisons between each risk profile and HC. No global differences were found in mean CT, or evidence for regions of shared cortical pathology in 22q11DS and UHR subjects.

### Global morphology indices: cSA, cGMV, and mean CT

22q11DS subjects had less cSA and cGMV compared to the UHR subjects. These findings are in line with previous studies comparing 22q11DS subjects to otherwise healthy subjects with sub threshold psychotic symptoms [12]. Another study also found these broad scale differences in cortical morphology in comparisons with HC subjects [14], suggesting these aberrations in cortical development to be specific for 22q11DS subjects. Reduced cSA and cGMV are also consistently reported in children and adolescents with 22q11DS in comparison typically developing subjects [15,36,37], showing these changes are already present in early cortical maturation and thus not likely the hallmark of defective pruning in adolescence. Interestingly, 22q11DS and UHR subjects seem to have normal maturation of mean CT in comparison to HC, suggesting that the overall modular buildup of the cortical layer is relatively unscathed by diagnosis in either risk profile. The comparable mean CT in 22q11DS subjects, UHR subjects and HC, implies that the lower cSA in the 22q11DS group was driven by lower amount of cortical surface embedded in the sulci–or lower LGI. In congruence our study and others report widespread lower LGIs in the prefrontal- and temporal gyri in comparison to HC subjects [12–14].

In line with other studies [17,18], the UHR subjects did not significantly differ in cSA, and cGMV from the HC suggesting that presence of attenuated psychotic symptoms is not related to pathology in the global outcome of cortical maturation.

### Vertex-wise comparisons of CT

Vertex-wise comparisons revealed that the UHR subjects were characterized by lower CT in the insula compared to the 22q11DS subjects, replicating earlier findings of lower insular CT in otherwise healthy subjects experiencing sub-threshold psychotic symptoms in comparison to 22q11DS subjects [12]. In comparisons with matched HC however, neither this study, nor ours, found evidence for abnormally thin CT of the insula in UHR subjects or individuals with sub-threshold psychotic symptoms [12]. Thus, cortical outcome in UHR subjects seems to be associated with CT values of the insula at the lower end of typical development and in 22q11DS subjects at the higher end of typical development. The insula has high rates of pruning during adolescence and a somewhat lower insula CT in UHR may stem from excessive pruning in adolescence and a somewhat higher insula CT in 22q11DS subjects may reflect delayed pruning. Taken together this region seems distinctive for each risk profile and thus, may be an interesting marker to follow up on longitudinally. Mal maturation of insula CT in adolescence may be critical to developing psychotic symptoms.

The cortical layer of the insula harbors spindle neurons in the lamina V layer that thus far have only been found in the insula, anterior cingulate cortex, and the dorsolateral prefrontal cortex [38]. These neurons are particularly important for regulation of self- awareness and social cognition [38]. Presence of social cognitive deficits and reduced self-awareness are prognostic symptoms in UHR subjects for the development of psychosis [39]. Lower CT in the insula may be related to insufficient maturation or defective pruning of cortical layers, including lamina V. UHR subjects may thus be more vulnerable to developing disturbed regulation of self-awareness and social cognition compared to 22q11DS subjects. In order to investigate spindle neurons in the insula and their role in the development of psychosis, a different MRI imaging technique would need to be employed. Advances in high field (7 Tesla) functional and structural MRI allow for layer specific assessments of the cortical mantel, and could be used to investigate underlying mechanisms of cortical risk factors [40,41].

The current study found no evidence for shared CT pathology between UHR and 22q11DS subjects in comparisons with HC. We did not replicate earlier findings identifying higher superior frontal gyrus CT as shared neuro-correlate [12] between healthy subjects with sub threshold psychotic symptoms and 22q11DS subjects. No other studies report increased CT of superior frontal gyrus in adult 22q11DS subjects, although it has been reported in adolescents with 22q11DS [13]. In UHR subjects a substantial amount of findings actually point towards the opposite pattern to reduced fronto-temporal CT [17,18,42], rather than increased CT. Uncorrected findings in the current study also point to lower CT in the bilateral superior and rostral middle frontal, inferior temporal in UHR subjects in comparison with HC subjects. Although there is a chance of a type II error, these regions are highly comparable to other studies [17,18,42], suggesting that these regions may mature differently in clinical and genetic risk groups for psychosis.

### Comparisons in Gyrification (LGI)

Our results showed that the 22q11DS subjects were characterized by lower LGIs of the *bilateral* prefrontal cortex, precentral gyrus, precuneus, the *right* superior and inferior temporal cortex, and the *left* fusiform gyrus compared to UHR subjects (see Fig 2). The study by Schmitt et al., investigating 22q11DS subjects to healthy subjects with sub-threshold psychotic symptoms identified very similar regions to have lower LGI [12], suggesting that lower LGI in these regions is a specific trait for 22q11DS.

Other studies in 22q11DS also report abnormal LGI in these regions, with one longitudinal study showing absence of the normal rate of change in LGI, particularly in the frontal cortex [13,14]. Cortical gyrification is largely complete prenatally, with only postnatal increases in in the superior and inferior frontal gyri [10]. Thus findings to date suggest both malformation and malmaturation of cortical gyrification in 22q11DS that is not reported in UHR subjects, nor reported by the current study. Studies investigating first episode psychosis patients reported hypogyrification (lower LGI) in regions of the frontal lobe in comparison to siblings, and healthy controls [22,43]. Moreover, lower LGI in fronto-insular and fronto-temporal regions was found predictive of non-response to antipsychotic treatment [44]. Potentially, the degree of frontal gyrification is related to the increased risk to develop a psychotic disorder in 22q11DS. To date, no studies including the current study, have reported lower LGI of the frontal-temporal regions to dissociate between 22q11DS subjects that have a positive history for psychosis and those that do not, although no longitudinal assessments have been made and groups tend to be too small to make inferences. Further longitudinal investigation needs to be done to assess whether maturation of frontal gyrification is a genetic risk factor in 22q11DS subjects.

Changes or mal-development of white matter underlying of the cortical mantel, particularly of interhemispheric connections, has been related to lower LGIs [10]. The underlying mechanisms of lower LGI in 22q11DS are unclear, although mouse models reveal that diminished dosage of 22q11.2 genes disrupt proliferation of basal progenitors, leading to an altered frequency of lamina layer II and III of the cortex, leaving layers V and VI unaffected [45]. Neurons in lamina layers II and III form the interhemispheric connectivity of the brain [46]. Investigation of differences in white matter microstructure between 22q11DS and UHR subjects conducted by our research group highlighted that 22q11DS subjects, but not UHR subjects were characterized by lower axonal integrity of the large inter-hemispheric cortical fasciculi [47]. As increases in LGI are driven by axonal fiber tension drawing more densely interconnected regions closely together, lower fiber tension and dysconnectivity may underlie the hypogyrification and cSA loss in 22q11DS subjects.

The current study reported no regions of lower LGI in UHR compared to 22q11DS, nor in comparisons to HC subjects. A single study has reported hypogyrification in the superior temporal gyrus and ACC in subjects [20], with a first degree relative with a psychotic disorder, and thus with an increased genetic load. Results thus, seem to point to genetic effects influencing gyrification, and clinical risk factors to aberrations in CT.

Taken together, UHR subjects showed no signs of early neurodevelopmental pathology of the cortex in gyrification, but showed signs of mal maturation of CT in the insula. Thinner insular CT has also been a robust finding in first episode of psychosis subjects [1,17,18] and may be distinctive for the more clinical pathway to psychosis. In contrast, 22q11DS subjects showed more prominent effects of early cortical gyrification pathology with widespread lower LGIs throughout the cortex. Hypogyrification in 22q11DS subjects could possibly be related to loss of underlying connectivity supporting gyrification, rather than CT, that more typifies the UHR subjects.

### Correlations between PANSS scores and morphometric indices

**Fig 3:**
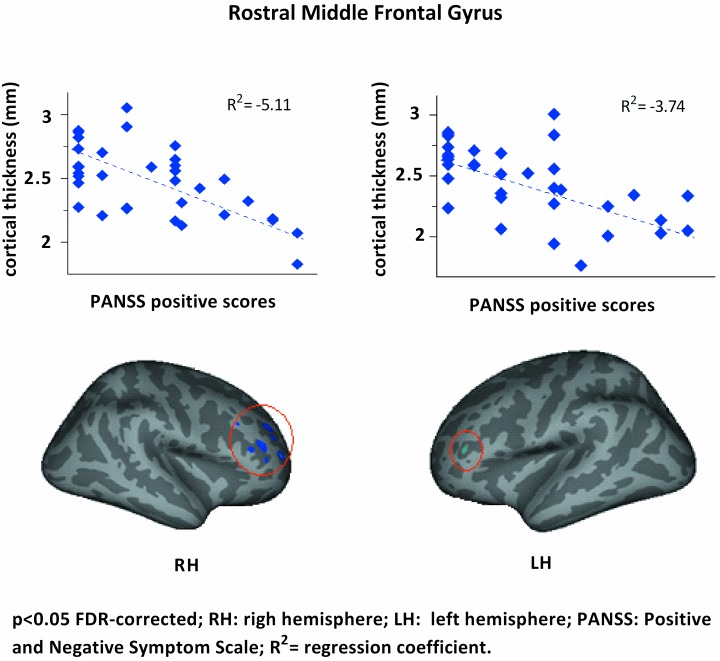
Bilateral thinner rostral middle frontal gyrus CT was associated with increased positive symptoms severity in both UHR subjects (n = 18) and matched 22q11DS (n = 18) subjects. Findings were corrected for differences in brain segmentation volume, age and gender. See S1 and S2 Files.

Lower rostral middle frontal gyrus CT was related to increased positive symptom severity (Fig 3) in both risk groups on a whole. Shared vulnerability to develop psychotic disorders may in part be related to sub-optimal development of the CT in this region. A previous longitudinal assessment in 22q11DS subjects showed development of psychosis was related to normalization of abnormally low middle frontal gyrus CT combined with more extensive frontal hypo-gyrification [13], although this study was underpowered with only 6 subjects developing a psychotic disorder. Thus, our results may underestimate CT of the middle frontal gyrus and overestimate frontal hypo-gyrification in the 22q11DS group. However, in line with these longitudinal findings, our study also identifies lower CT in the middle frontal gyrus to be related to increase positive symptom severity. No clear relationship between psychotic symptom severity and CT in UHR subjects have been previously reported, potentially due to the fact that no longitudinal investigations have been done in UHR subjects on cortical morphology. No corrected group specific correlations were found between rostral middle frontal gyrus CT and positive symptoms severity although statistical power was likely to small to detect such a correlation. This would need to be investigated more conclusively in a larger sample. Potentially this is a shared neurocorrelate in the pathway to psychosis. Despite the aberrant prefrontal hypogyrification found in the 22q11DS subjects in the current study, and the comparable findings of prefrontal hypogyrification in patients with psychosis [48], we found no significant relation between LGI and psychotic symptom severity in 22q11DS subjects.

### Strengths and limitations

A strength of the current study is that it is the first to assess the finishing stage of cortical developmental in relation to clinical and genetic risk factors and psychotic symptoms in adult high-risk populations. In UHR subjects a comprehensive meta-analytical study found that risk rates for developing a psychotic disorder increased with age at which UHR status was identified [49]. Thus, the included adult UHR subjects assessed in the current study are likely to have higher risk rates than previous studies assessing cortical morphology in UHR. Additionally all, results discussed were found under stringent correction for multiple testing and performed using 3 Tesla images. Most former studies investigating cortical morphology in UHR were scanned at lower field strengths.

A limitation of the current study, however, was the significant difference in positive symptom severity between UHR and 22q11DS patients, which may in part explain differences in CT and LGI reported. Importantly, the groups were matched on negative symptom severity. Current literature shows that negative, rather than positive symptom severity, predict transition to psychosis [50,51]. Thus, our results are not biased by a possibly more frequent transition to psychosis in either group.

Changes in cortical morphology in 22q11DS have been proposed to stem from cortical hypo- perfusion due to presence of congenital heart disease. This potential confound was tested and we found no significant effects of congenital heart disease on any of the morphology indices, although we cannot rule out medicinal effects on cortical morphology in the 22q11DS subjects that developed psychosis.

A limitation of the current study, and of many other studies in the field, is the lack of longitudinal data to identify which progressive changes in UHR and 22q11DS are specific to those that will transition to psychosis. We need this information to render in risk factors that are better predictors of illness transition. As of yet, there is a particular lack of longitudinal studies investigating cortical morphology in UHR subjects from adolescent age to adulthood.

## Conclusions

In conclusion, despite a shared increased risk for developing psychotic disorders, our results suggest that the risk in 22q11DS subjects is deferred through early neurodevelopmental effects related to widespread hypogyrification, which potentially may be a marker of comprised underlying connectivity. In contrast, UHR patients were typified by regionally lower CT of the insula, with no indication of early neurodevelopmental pathology. In both risk populations, however, lower rostral middle frontal gyrus CT was related to increased positive symptom severity. It would be interesting to investigate changes over time of rostral middle frontal gyrus CT in UHR and 22q11DS subjects that clinically present with predominantly positive symptoms. In addition, to better understand later pathological processes of cortical maturation and the influence on psychotic symptoms, longitudinal studies should investigate changes in cortical morphology over time in both UHR and 22q11DS subjects.

## Supporting Information

S1 File Raw data output for cortical thickness and local gyrification indices. (XLSX)

S2 File General linear model design used for QDEC for comparisons between ultra-high risk subjects and 22q11DS subjects in cortical thickness and local gyrification. (DAT)

S3 File General linear model design used for QDEC for comparisons between healthy control subjects and 22q11DS subjects in cortical thickness and local gyrification. (DAT)

S4 File General linear model design used for QDEC for comparisons between healthy control subjects and ultra-high risk subjects in cortical thickness and local gyrification. (DAT)
